# Young Adults, Technology, and Weight Loss: A Focus Group Study

**DOI:** 10.1155/2015/379769

**Published:** 2015-02-18

**Authors:** Janna Stephens, Gyasi Moscou-Jackson, Jerilyn K. Allen

**Affiliations:** Johns Hopkins University School of Nursing, 525 N. Wolfe Street, Baltimore, MD 21205, USA

## Abstract

Overweight and obesity are a major concern in young adults. Technology has been integrated into many weight loss interventions; however little is known about the use of this technology in young adults. The purpose of this study was to explore through focus group sessions the opinions of young adults on the use of technology for weight loss. A total of 17 young adults, between 18 and 25 years of age, participated in three focus group sessions. Major results indicated that young adults have very little knowledge on the use of Smartphone technology for weight loss but would like to use this type of technology to help them lose weight. Results also indicated that young adults struggle to make healthy food choices and have priorities that outweigh exercise and they need support and guidance to make better decisions. In conclusion, young adults would be open to using Smartphone technology for weight loss but also need feedback and guidance to help make healthy decisions.

## 1. Introduction

Overweight and obesity are a major concern for young adults in the United States. Young adults are at high risk of being overweight and obese and many begin to gain weight when they enter the college [1]. Studies have indicated that over 29% of young adult males and females, aged 20–34 years, are overweight or obese [2]. Among college freshmen, many gain weight and continue to gain a significant amount of weight through their senior year [3]. Being overweight or obese can lead to complications later in life, such as diabetes, heart disease, sleep apnea, hypertension, elevated cholesterol, and even death [4–6].

Targeting college-aged individuals is important because college is a time where many lifestyle changes occur. Individuals can make their own food choices without influence of family and are subjected to many unhealthy habits, such as excessive snacking, unhealthy meal choices, and busy schedules leading to a decrease in time spent in physical activity. Qualitative studies have reported many factors related to weight gain in college students, including late night snacking, poor time management, stress eating, unhealthy food availability on campus, and lack of motivation [7–9]. There is a need to understand the types of interventions that would target college students who are interested in losing weight.

Existing literature reports that weight loss and other healthy habits can be achieved in young adults through various interventions. One study utilized Facebook with a personalized messaging component and saw significantly greater weight loss in this group versus control (−2.4 kg versus −0.24 kg; *P* < 0.05) [10]. An additional study tested an Internet based obesity prevention program designed for college students. The study reported an increase in fruit and vegetable consumption and a decrease in stress compared to control, although they did not report any differences in exercise behavior or weight loss [11]. A study utilizing behavioral self-regulation reported significant weight loss in two groups receiving intervention (−6.6 kg and −5.8 kg, *P* < 0.001). A study done by LaRose et al. [12] tested two interventions in young adults. One group was assigned small changes (SC), in which changes in energy balance were small (200 kcal/day). The second group was assigned large changes (LC) and was instructed to cut 500–1000 calories for daily intake. Weight changes were significantly different between the two groups at both 8 weeks (SC = −0.68 ± 1.5 kg, LC = −3.2 ± 2.5 kg, and *P* < 0.001) and 16 weeks (SC = −1.5 ± 1.8 kg, LC = −3.5 ± 3.1 kg, and *P* = 0.006) [12].

Incorporating technology into weight loss interventions has proven to be successful [13]. Smartphone technology can provide an individual with the ability to self-monitor diet and physical activity, receive feedback through the application or text messaging, and track progress towards goals. However, there is limited knowledge on the use of Smartphone technology for weight loss in young adults. In particular, the young adult's perspective on weight loss interventions that include a technology component has not been explored. When introducing new technology into interventions, it is critical to explore the opinion of the recipient. Therefore, the purpose of this study is to explore a young adult's perspective on nutrition, exercise, and technology for weight loss in order to design a randomized controlled trial.

## 2. Materials and Methods

This study used a qualitative exploratory design with focus group sessions. It was approved by the Johns Hopkins Medical Institutional Review Board (IRB).

Young adults were recruited through posters and flyers posted around undergraduate and graduate campuses. A brief description was also posted on a university's online portal that students can view when logging into their email or other university related places. Word of mouth was also a popular recruitment method for the study. In order to participate, individuals had to be between the ages of 18–25 and had to be interested in weight loss. Young adults were not required to be overweight to be in the study; the goal was to gain the perspective of students who wanted to lose weight as well as those wanting to prevent weight gain.

Three focus group sessions were conducted between June 2013 and July 2013 by one researcher acting as the primary moderator. All focus groups were conducted in a casual environment in a private room of a campus library. The sessions each had one moderator and one note-taker. The note-taker's purpose was to watch for body language, facial expressions, and group reactions that could not be picked up on the tape recordings. The note-taker and moderator met after each focus group meeting to discuss the notes that were taken. Each session lasted approximately one hour. All sessions included between 5 and 7 young adults. A total of 17 young adults were included in the three focus groups.

All sessions were tape-recorded. Three handheld tape recorders were used to record each session to ensure that all comments were recorded clearly and accurately. Demographic information, including age and race, was collected from each young adult at the beginning of each session. The moderator introduced herself and gave a brief explanation of the procedures to the focus group session along with a description of the focus group study. Written informed consent was obtained individually from young adults. Each young adult received a $25 gift card for his or her time.

A single discussion guide was created prior to the focus group meetings. This guide was meant to introduce topics to young adults with open-ended questions, as well as asking minimal directive questions (see Table 1). The group discussions focused on the following topics: overweight and obesity in young adults, diet and exercise habits, counseling strategies for weight loss or weight maintenance, the use of Smartphone technology for weight loss, and texting/email feedback.

The moderator emphasized that all responses were welcomed and that no response was considered right or wrong. Quiet young adults were periodically asked if they had anything to share. Open-ended questions were used to begin each section of the discussion (“Tell me your thoughts on…”). The questions became more focused once the topic was introduced (“What specific topics would you want to discuss when talking with someone about weight loss…”). Table 1 describes the discussion guide and example questions from each topic.

Qualitative analysis of each focus group session was done using a process of thematic analysis. The process began with an initial read of the transcribed data and reading of the notes taken during the session. The analysis procedure consisted of multiple readings of the transcripts to identify key words or phrases. Coding began during the second read of the data. The relevant passages were coded initially using words from the young adults themselves. Subsequent readings of the transcripts and the passages coded in similar ways yielded categories of data describing the critical issues for young adults. Categories from each topic were combined to form themes, which provided a comprehensive view of the data.

## 3. Results

A total of 17 young adults were included in the three groups. Ages ranged from 18 to 25 years (mean = 21 years). Across all focus groups 59% were female, 29% were African American, and 24% were white. In this study, 5 young adults wanted to maintain weight and 12 wanted to lose weight. The results across focus groups are organized by major themes with a general explanation of findings and exemplar quotes.

### 3.1. Theme: Freedom to Choose Often Equals Overweight and Obesity

Across all focus groups, young adults expressed that overweight and obesity among young adults in the United States are a major issue. It was particularly clear that those who migrated to the United States in the past ten years saw overweight and obesity as a larger problem than those that had lived in the United States the majority of their lives. However, those that had been in the United States for most of their lives still identified weight as an increasing problem for young adults. Several potential causes of overweight and obesity among young adults were identified. The responsibility on the young adult to make healthy food choices was identified as the primary cause for weight gain in this population. Young adults expressed that having the ability to make choices, free of family influence, made it more difficult to choose healthy foods, especially because fast and convenient foods are so readily available. Also, for those in college, stress eating was identified as another major cause of overweight and obesity. Young adults expressed that attending classes, not sleeping, having to prioritize activities, and time management all lead to stress eating.

*“It's a lot different than your life as a high school student, the stress and the new schedule of classes add to the pressure of how you eat and if you workout [sic] or not.”*

Alcohol was mentioned as another reason why many college students gain weight. The freedom of living alone, without parents, creates a situation in which individuals can consume alcohol freely. It is clear from the groups that many young adults engage in activities that involve alcohol both during the week and on the weekend.

*“My roommate last year, he tried to lose weight and he would run like five miles a day but he would also drink…umm and then he would never really like lose that much weight because he drank too much.”*

### 3.2. Theme: Priorities Are Key

Young adults discussed what types of diet and physical activity habits they currently have and what influences those habits. They primarily expressed eating foods that are considered convenient, such as food from fast-food restaurants or food readily available in school cafeterias. The majority of young adults in the study were college students and explained that a lack of time to prepare meals was an important factor when making food decisions. Any free time they have is spent doing more important tasks. These young adults tended to choose foods that were cheap because most were living on a budget or they simply ate in the school cafeterias because they had a meal plan. Other competing priorities also get in the way of making healthy food choices. For example, choosing sleep over eating breakfast was a common occurrence in this population. The focus groups also highlighted that young adults lack knowledge about sugar-sweetened beverages and because they lack knowledge, they tend to consume very high amounts of these beverages.

*“I feel like there is not much difference between the diet ones [soda] and the regular ones…. I mean like the calories or whatever.”*

In terms of looking at the labels on sugar-sweetened beverages, many young adults look at the label but do not considerate it when making choices.

*“I look at it but I guess I consider the food portion more than the beverage calorie portion, which doesn't make any sense, but just psychologically, like of course you're eating so of course you're going to consider those calories more than what you are drinking.”*

Activity levels in young adulthood depend on the individual, some increase activity, and others decrease activity levels from high school. Many young adults especially those in a college atmosphere have access to free workout facilities. However, their busy schedules and lack of sleep often compete with their drive to workout. Young adults emphasized the need and want to make exercise a social activity and if their friends do it, they will do it. However, like with diet, they have priorities that constantly compete with the ability to find time for physical activity such as sleep, studying, classes, and hanging out with friends.

### 3.3. Theme: Squash the Rumors and Talk with an Expert

Following the discussion of nutrition and physical activity habits, the young adults were asked about what information they would want to receive in a counseling session regarding diet and physical activity. All of the young adults identified diet as the most important part of any counseling session. Many young adults believe in the “80/20” theory, that weight loss is 80% diet and 20% physical activity. Knowing and understanding how much food they should be consuming were the greatest need, as most did not know how many calories they should be consuming per day to lose weight. Another major concern for young adults was weight maintenance and learning how to keep weight off once they lose it. Young adults would also like help defining reasonable goals. They want help from an expert and specifically want to learn how to work towards and achieve those goals by eliminating or getting through barriers. All young adults are exposed to many rumors about what works and what does not, as well as different fad diets. They would like knowledge about these types of diets and are interested in learning what is fact versus what is fiction. In terms of exercise, young adults expressed wanting to learn about types of exercise that are best for weight loss. They also wanted to learn about heart rates, calories burned during exercise, distance exercising, and how to pull all of this information together for use during their workouts.

### 3.4. Theme: Give Me an App That Does It All

The major finding from the focus group discussion is that very little is known about what is available for use regarding Smartphones and applications for weight loss. Most of the young adults identified that they would want an application that could track diet and exercise, track weight changes, had an easy way to enter foods and exercise, and could tell them the calories in restaurant foods.

*“And I was thinking, if they could come up with an app that could umm give suggestions…like once you put your weight and age in, it would come up with at least how many walks you should have everyday, how much water you should drink everyday….”*

*“I kind of want it to have a calorie input thing, so that I can put how many calories I had that day.”*

Very few young adults knew that any of these features were available in current Smartphone applications and when they heard that it was available, they were excited to use this type of technology. The demand for an individualized program was high. Young adults wanted an application that is specific to their height, weight, gender, age, and weight loss goals. They also expressed interest in applications that have features that make tracking easy, such as a barcode scanner which could scan and autoenter nutritional information from the foods they have consumed. When asked about a barcode scanner, a young adult replied, “that would be the coolest thing ever.” The application should also contain a large database of restaurants and premade foods, so that young adults are not guessing on the amount of calories in foods or relying on the restaurant menu to contain this piece of information. The main finding from this section was that young adults wanted to use this type of technology but did not realize that it was available for their use.

### 3.5. Theme: Personalize All Messages or Risk Being Ignored

The final area assessed was the type of feedback young adults would want to receive from a health coach and what timing and mode of information delivery would be best (e.g., text message or email). Young adults stressed that any messages received, whether text messages or emails, must be individualized. They did not want to receive standard messages that are sent out to a group or automated from a system. Young adults expressed that they tend not to read generic messages, such as those they often receive from their university regarding safety concerns. Further, messages sent via text or email should be specific to a young adult's progress in a weight loss program, meaning that the health coach should pay close attention to areas where goals are being met and areas where improvements could be made.

*“I think it would be good, as long as it wasn't generic, because if it was generic, I would probably not pay attention to it.”*

*“I would want them to be pretty personal. Like if you saw that I was not doing something, then the message would be encouraging to what I was not doing, or something like that.”*

There is great variation in the number of messages that young adults expressed wanting when working with a health coach. Many young adults wanted at least one message per day, focused around meal times. Other young adults wanted only 1-2 messages per week, containing more summary information to guide their progress. The consensus was that once per week is a minimum for contact during a time when a young adult is trying to lose weight.

Information that should be contained in the messages included reminders to log foods or exercise into an application or diary. Also, encouragement to meet preestablished goals was important to young adults. One important factor to consider when sending a specific and individualized message is that young adults expressed not wanting any negative feedback sent to them. For example, if the healthcare provider or health coach is monitoring foods logged, young adults do not want to be “scolded” for eating something like a “cupcake.” Messages should instead be framed as suggestions of healthy, nutritious foods to consume and ways to possibly cut back on less nutritious foods. Receiving healthy recipes was also of great interest, as long as the recipes are simple and easy to follow. As a follow-up to the more individualized messages, young adults were all interested in receiving a summary statement weekly, something to show how they ate overall for the week, how they exercised for the week, and any progress made for the week.

## 4. Discussion

The results of this study should inform the development of weight loss or weight maintenance interventions in young adults, particularly college students. Five themes were assessed during this study: (1) freedom to choose often equals overweight and obesity, (2) priorities are key, (3) squash the rumors and talk with an expert, (4) give me an app that does it all, and (5) personalize messages or risk being ignored. There were several key take-home messages related to the specifics of weight loss noted in this study as follows.

- Young adults are excited to use applications to help them lose weight.
- Young adults often gain weight due to freedom to make food choices.
- Time is a priority and often competes with weight loss.
- Messages should contain individual feedback.
- Motivational and positive feedback messages are preferred.
- An application that is all-inclusive is desired.

Regarding overweight and obesity, young adults expressed that this is a frequent problem in this population, which they attribute to newfound independence. This independence leads to making food and cooking decisions without the influence of family. Therefore, similar to other weight loss intervention studies in adults, a weight loss intervention for young adults should include a counseling session that includes tools for increasing knowledge and promoting good decision-making. This decision-making should include information on what makes up a healthy plate and how to make good choices even when faced with convenient, unhealthy foods. Young adults who are in college should receive counseling on the nutritional information of food choices provided in school cafeterias and how to make the best choice in a situation where there might not be a very healthy option. Also, as alcohol seems to play a role in weight gain in this population, counseling on the caloric intake of alcoholic drinks should be included along with strategies to consume fewer calories from alcohol. In a weight loss trial in young adults, a counseling session should also include time to learn the young adult's motivations for weight loss and priorities that will compete with the ability to lose weight. Working through barriers and setting goals is important in this population and should be a major focus.

Young adults expressed that they knew that diet and physical activity were important, but their habits were heavily influenced by time and cost. They had several competing priorities that inhibited their ability to choose nutritious foods so they resorted to fast and convenient foods or sometimes skipped meals like breakfast altogether. Despite knowing that diet and physical activity were important, young adults may have a hard time determining which foods to consume or avoid and which exercises are best. The lack of knowledge about SSB, nutrition fads, and breakfast consumption was evident. Therefore, a discussion and session on diet fads, SSB consumption, breakfast consumption, and more should be included when working with young adults who wish to lose weight.

As far as the authors know, this is the first focus group study to explore the use of Smartphone technology for weight loss among young adults. What was evident from the focus group is that young adults lack awareness of the Smartphone technology that exists to help with weight loss. Young adults were unaware of what types of weight loss applications are available and they showed a lot of interest in learning about them and using them in their future. Results also suggest that young adults may benefit from features such as using an application to set individualized calorie budgets and goals. Time to input food and exercise is an issue for young adults; however, many applications have features such as barcode scanners, restaurant menus, and custom exercises that make logging faster and easier. An application that has these features may be used more readily in the young adult population.

There was little consensus on the frequency of contact via text messages, but once per week was an agreed minimum and three times per day seemed to be an agreed maximum. Young adults did not come to a consensus on time of day to receive messages and this varied from 7 am to 12 am. Young adults agreed that contact should be individualized and positive. Text messages cannot be automated messages and need to come from a person who acts as a health coach or motivator to the individual. It is clear from the results of this study that young adults need to have the ability to choose the amount of messages they wish to receive per day and they should also choose the method (email or text) as well. Also, when working with young adults, monitoring of their food and exercise should be used as a way to form motivational messages to send; this way the messages are more personal and directly relate to their daily habits. This way, the young adult stays engaged and is receiving the type of contact that will best help them reach their goals.

## 5. Conclusion

This focus group study explored the young adult's opinion on overweight/obesity, counseling for weight loss, and the use of Smartphone technology. It is clear from the study that young adults who are interested in weight loss are open and excited about using applications. Although young adults do not know about specific technology that exists, they are open to learning this technology as long as it fits into their lifestyle.

## Figures and Tables

**Table 1:** Discussion guide questions.

Topic	Opening question	Follow-up question	Follow-up question	Follow-up question
General feelings on overweight/obesity in young adults	I would like to hear about your thoughts on overweight and obesity in the young adults.	Have you ever heard that college is a time where people gain weight?	Do you know many people your own age who are trying to lose weight?	How healthy do you think young adults are in general?

Counseling session	I would like to hear your thoughts on what types of information you would want to discuss with someone if you were trying to lose weight.	Can you describe what you think about exercise when it comes to losing weight?	What are your thoughts on sugar-sweetened beverages and weight loss?	Do you see eating breakfast as something important for a person trying to lose weight? Please explain.

Smartphone application/text and email contact	What types of things would you want a Smartphone application to have to help you lose weight?	How would you feel about messages to help keep you on track?	What types of messages would keep you motivated? Can you give an example of what one might say?	What do you think is a good amount of messages? How many messages would you want to receive?
